# Flg22-Triggered Immunity Negatively Regulates Key BR Biosynthetic Genes

**DOI:** 10.3389/fpls.2015.00981

**Published:** 2015-11-09

**Authors:** Tamara Jiménez-Góngora, Seong-Ki Kim, Rosa Lozano-Durán, Cyril Zipfel

**Affiliations:** ^1^The Sainsbury Laboratory, Norwich, UK; ^2^Shanghai Center for Plant Stress Biology, Shanghai Institutes for Biological Sciences, Chinese Academy of Sciences, Shanghai, China; ^3^Department of Life Science, Chung-Ang University, Seoul, South Korea

**Keywords:** immunity, brassinosteroids, crosstalk, transcriptional regulation, flagellin

## Abstract

In plants, activation of growth and activation of immunity are opposing processes that define a trade-off. In the past few years, the growth-promoting hormones brassinosteroids (BR) have emerged as negative regulators of pathogen-associated molecular pattern (PAMP)-triggered immunity (PTI), promoting growth at the expense of defense. The crosstalk between BR and PTI signaling was described as negative and unidirectional, since activation of PTI does not affect several analyzed steps in the BR signaling pathway. In this work, we describe that activation of PTI by the bacterial PAMP flg22 results in the reduced expression of BR biosynthetic genes. This effect does not require BR perception or signaling, and occurs within 15 min of flg22 treatment. Since the described PTI-induced repression of gene expression may result in a reduction in BR biosynthesis, the crosstalk between PTI and BR could actually be negative and bidirectional, a possibility that should be taken into account when considering the interaction between these two pathways.

## Introduction

Plants need to integrate multiple environmental signals to finely regulate their growth and development in an adaptive manner. Activation of growth and activation of defense against potential pathogens are opposing processes, and the onset of one frequently results in inhibition of the other (Belkhadir et al., 2014; Huot et al., 2014). In the absence of pathogen challenge, growth is prioritized over defense; upon detection of a pathogen, defense responses are initiated, at the expense of growth. This trade-off between growth and defense is regulated at multiple levels, and its control has been shown to depend on the action of several plant hormones, including jasmonates, gibberellins, brassinosteroids (BR), and salicylic acid (Navarro et al., 2008; Albrecht et al., 2012; Belkhadir et al., 2012; Yang et al., 2012; Lozano-Durán et al., 2013; Chandran et al., 2014; Fan et al., 2014; Malinovsky et al., 2014).

The first layer of plant defense relies on the perception of conserved pathogen-associated molecular patterns (PAMPs) by pattern-recognition receptors (PRR) at the cell surface (Boller and Felix, 2009; Macho and Zipfel, 2014). Recognition of a PAMP by the cognate PRR initiates a signaling cascade that involves signal transduction from the plasma membrane to the nucleus, where transcription is heavily reprogrammed. PAMP-triggered signaling ultimately leads to the activation of the so-called PAMP-triggered immunity (PTI), which is sufficient to ward off most potential pathogens. Activation of PTI also results in a strong inhibition of growth, which can be easily detected when seedlings are treated with certain PAMPs (Gomez-Gomez and Boller, 2000; Kunze et al., 2004). Conversely, the growth-promoting hormones BR can inhibit PTI: activation of BR signaling, triggered by exogenous hormone treatments or by genetic overexpression or activation of components of the pathway, results in a suppression of several PTI responses in *Arabidopsis* (Albrecht et al., 2012; Belkhadir et al., 2012; Lozano-Durán et al., 2013; Fan et al., 2014; Malinovsky et al., 2014). Since activation of PTI was not found to affect the BR signaling pathway, the BR-PTI crosstalk was described as unidirectional and negative (Albrecht et al., 2012; Belkhadir et al., 2012).

In this work, we describe that activation of PTI by application of the bacterial PAMP flg22 results in the reduced expression of BR biosynthetic genes. This effect can be detected 15 min after treatment, and is sustained during a 24-h treatment. Moreover, this reduction of transcript levels does not require BR perception or signaling. Because the observed PTI-induced repression of gene expression may result in a decrease in BR biosynthesis, the crosstalk between PTI and BR could actually be indirect, negative, and bidirectional, a possibility that should be contemplated when considering the interaction between these two pathways.

## Results

### Flg22 Treatment Results in the Repression of BR Biosynthetic Genes

**FIGURE 1:**
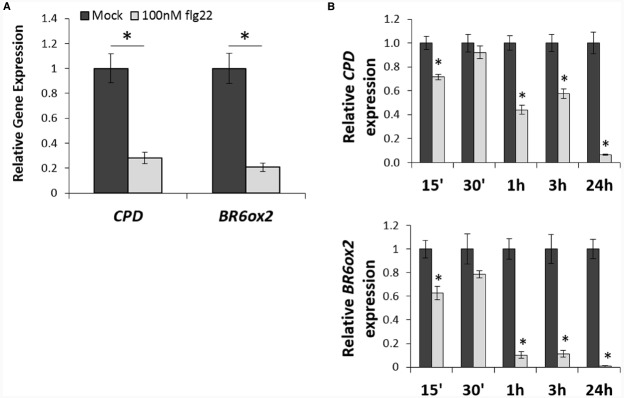
**Flg22 treatment downregulates expression of BR biosynthetic genes. (A).** Relative expression of the BR biosynthetic genes *CPD* and *BR6ox2* following treatment with flg22, as measured by qPCR. Ten-day-old seedlings (Col-0 wild-type) were submerged in a 100 nM flg22 or mock solution for 1 h. Bars represent standard deviation, with *n* = 3. Asterisks indicate a statistically significant difference, according to a Student’s *t*-test, with *p* < 0.05. This experiment was repeated three times with similar results; values from one representative experiment are shown. **(B)** Relative expression of the BR biosynthetic genes *CPD* and *BR6ox2* at different time points following treatment with flg22, as measured by qPCR. Ten-day-old seedlings (Col-0 wild-type) were submerged in a 100 nM flg22 or mock solution and samples were taken at 15 min, 30 min, 1 h, 3 h, and 24 h. Bars represent standard deviation, with *n* = 3. Asterisks indicate a statistically significant difference, according to a Student’s *t*-test, with *p* < 0.05. This experiment was repeated three times with similar results; values from one representative experiment are shown.

Activation of PTI induced by treatment with the bacterial PAMP flg22 leads to heavy transcriptional reprogramming in plants (Navarro et al., 2004; Zipfel et al., 2004). As part of these transcriptional changes, we observed that flg22 treatment consistently results in down-regulation of the BR marker gene *CPD*, which encodes a protein involved in BR biosynthesis (Szekeres et al., 1996; Table 1). An interrogation of publicly available microarray data (AtGenExpress collection; Schmid et al., 2005) revealed that flg22 treatment triggers a repression of several BR biosynthetic genes other than *CPD*, namely *DWF4*, *BR6ox1*, *BR6ox2*, *CYP90C1*, *BAS1*, *SMT2*, *DWF1*, and *DWF7* (Table 1). Down-regulation of a subset of these genes can also be detected upon treatment with other PAMPs (Tables 1 and 2), although to a lesser extent. For further analyses, *CPD* and *BR6ox2*, which are repressed in response to both flg22 and elf18 (Tables 1 and 2), were selected as marker genes, and their repression following flg22 treatment could be confirmed by qPCR (Figure 1A). A time-course analysis, depicted in Figure 1B, revealed that down-regulation of *CPD* and *DWF4* upon flg22 treatment can be detected 15 min after treatment, and is maintained over a 24-h treatment.

**TABLE 1:** **Expression changes in BR biosynthetic genes in response to treatment with different PAMPs**.

**Gene**		**flg22**	**flg22**	**HrpZ**	**HrpZ**	**LPS**	**LPS**	**1 μM**	**1 μM**
		**1 μM 1 h**	**1 μM 4 h**	**10 μM 1 h**	**10 μM 4 h**	**100 μg/ml 1 h**	**100 μg/ml 4 h**	**GST-NPP1 1 h**	**GST-NPP1 4 h**
***DWF4***	***At3G50660***	0.69	1.02	0.61	0.74	0.91	1.31	0.57	0.87
***CPD***	***At5G05690***	0.65	0.56	0.7	0.46	1.21	0.95	0.94	0.48
***DET2***	***At2G38050***	1.12	1.06	1.20	0.96	1.21	0.87	1.20	0.74
***BR6ox1***	***At5G38970***	0.38	0.71	0.87	0.90	0.56	0.48	2.90	0.90
***BR6ox2***	***At3G30180***	0.74	0.52	0.46	0.49	1.08	0.89	1.04	0.62
***CYP90C1***	***At4G36380***	0.72	1.02	0.77	0.7	0.90	1.23	0.71	0.45
***CYP90D1***	***At3G13730***	1.14	0.80	1.47	0.6	1.01	1.08	1.20	1.11
***BAS1***	***At2G26710***	0.6	0.42	0.55	0.42	2.54	0.71	2.65	0.28
***UGT73C5***	***At2g36800***	1.13	0.77	0.57	1.09	0.47	1.29	0.73	0.77
***SMT2***	***At1G20330***	0.61	0.82	0.63	0.87	0.86	1.02	0.77	0.83
***DWF1***	***At3G19820***	0.82	0.53	0.82	0.56	1.18	0.96	0.87	0.43
***DWF7***	***At3G02580***	0.78	0.66	0.99	1.03	0.99	1.03	0.83	0.63
***DWF5***	***At1G50430***	1.12	0.65	1.11	0.81	0.99	0.83	1.02	1.02

Transcriptional changes of BR biosynthetic genes in response to treatment with different PAMPs (flg22, HrpZ, LPS, GST-NPP1) (eFP browser). Values represent fold-increase, as compared to controls. Values below 0.80 (indicating downregulation of expression) are highlighted in blue; values over 1.20 (indicating upregulation of expression) are highlighted in yellow.

**TABLE 2:** **Expression changes in BR biosynthetic genes in response to treatment with elf18**.

**Gene**		**elf18 30 min**	**elf18 60 min**
***DWF4***	***At3G50660***	0.90	0.70
***CPD***	***At5G05690***	0.70	0.60
***DET2***	***At2G38050***	0.80	0.70
***BR6ox1***	***At5G38970***	NA	NA
***BR6ox2***	***At3G30180***	0.60	0.50
***CYP90C1***	***At4G36380***	NA	NA
***CYP90D1***	***At3G13730***	1.10	0.90
***BAS1***	***At2G26710***	NA	NA
***UGT73C5***	***At2g36800***	NA	NA
***SMT2***	***At1G20330***	0.90	0.80
***DWF1***	***At3G19820***	1.10	0.90
***DWF7***	***At3G02580***	1.00	0.90
***DWF5***	***At1G50430***	1.00	1.00

Transcriptional changes of BR biosynthetic genes in response to treatment with elf18 (Zipfel et al., 2006). Values represent fold-increase, as compared to controls. Values below 0.80 (indicating downregulation of expression) are highlighted in blue.

### The flg22-mediated Repression of BR Biosynthetic Genes is Independent of BR Perception

**FIGURE 2:**
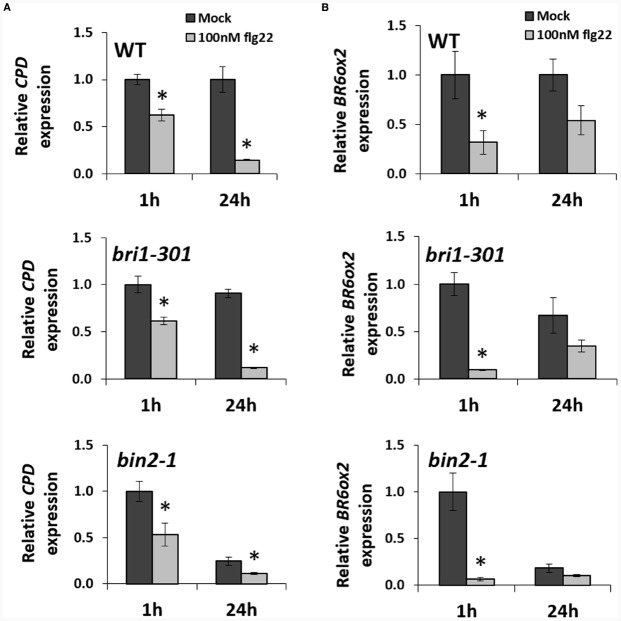
**The flg22-triggered repression of BR biosynthetic genes does not require BR perception or signaling.** Relative expression of the BR biosynthetic genes *CPD* **(A**) and *BR6ox2* **(B)** in *Arabidopsis* Col-0 wild-type (WT) and the BR signaling mutants *bri1-301* and *bin2-1*, following treatment with flg22, as measured by qPCR. Ten-day-old seedlings were submerged in a 100 nM flg22 or mock solution for 1 or 24 h. Bars represent standard deviation, with *n* = 3. Asterisks indicate a statistically significant difference, according to a Student’s *t*-test, with *p* < 0.05. This experiment was repeated three times with similar results; values from one representative experiment are shown.

Because expression of BR biosynthetic genes is subjected to a negative feedback loop, and therefore these genes are repressed upon activation of BR signaling (Bancos et al., 2002; He et al., 2005; Tanaka et al., 2005; Sun et al., 2010; Yu et al., 2011), we wondered whether the observed flg22-triggered repression of *CPD* and *BR6ox2* required BR signaling. In order to determine this, we probed the expression changes of these two genes in the BR signaling mutants *bri1-301*, impaired in BR perception, and *bin2-1*, in which BR signaling is disrupted downstream of BR perception and upstream of BR-induced transcriptional changes (Peng et al., 2008; Xu et al., 2008). In both mutants, repression of *CPD* and *BR6ox2* could be detected after 1- or 24-h flg22 treatments (Figures 2A,B), indicating that these expression changes do not require an intact BR signaling pathway.

**FIGURE 3:**
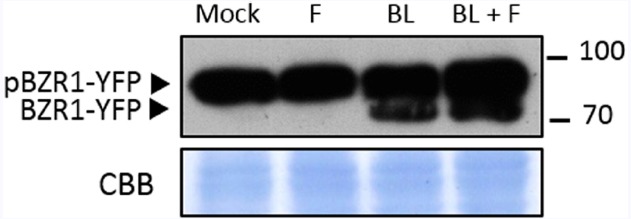
**Treatment with flg22 does not affect phosphorylation of BZR1.** Accumulation of BZR1-YFP in its phosphorylated (pBZR1-YFP) and de-phosphorylated (BZR1-YFP) forms upon treatment with flg22 (F), brassinolide (BL), flg22 and brassinolide (F+BL) or mock solution. Ten-day-old transgenic *Arabidopsis* seedlings expressing BZR1-YFP were submerged in a 100 nM flg22, 1 μM brassinolide, 100 nM flg22 + 1 μM brassinolide or mock solution for 1 h. Total proteins were separated in a 10% acrylamide gel and transferred to a PVDF membrane. The membrane was blotted with anti-GFP antibody. CBB: Coomassie brilliant blue. This experiment was repeated four times with similar results.

Brassinosteroids signaling, as PTI signaling, leads to the transcriptional reprogramming of the cell (Nemhauser et al., 2006; Sun et al., 2010; Yu et al., 2011). The two major transcription factors mediating these changes are BZR1 and BES1 (Wang et al., 2002; Yin et al., 2002; Sun et al., 2010; Yu et al., 2011). Recently, BZR1 was described to mediate crosstalk between the BR and the PTI signaling pathways (Lozano-Durán et al., 2013); BES1 has also been proposed to interact with PTI responses (Kang et al., 2015). Both *CPD* and *BR6ox2* are targets of BZR1, which down-regulates their expression when activated (He et al., 2005; Sun et al., 2010); while *CPD* has also been found to be repressed by BES1, *BR6ox2* has not (Yu et al., 2011). The activation of BZR1 depends on its phospho-status, since only de-phosphorylated BZR1 is active (He et al., 2002; Zhao et al., 2002; Gampala et al., 2007; Ryu et al., 2007), as well as on the availability of interacting partners that act as transcriptional co-regulators (Luo et al., 2010; Bai et al., 2012; Gallego-Bartolome et al., 2012; Li et al., 2012; Oh et al., 2012). Upon activation of BR signaling following BR perception, BZR1 is rapidly de-phosphorylated (He et al., 2002; Tang et al., 2011). In order to determine if the flg22-triggered down-regulation of *CPD* and *BR6ox2* expression depends on BZR1, we investigated the phospho-status of this transcription factor (BZR1-YFP; Gampala et al., 2007), as a proxy for its activation status, in response to flg22. As shown in Figure 3, de-phosphorylated BZR1 can be detected following treatment with the BR brassinolide (BL), but not flg22; co-treatment with flg22 does not affect the effect of BL. Taken together, these results indicate that the flg22-triggered repression of *CPD* and *BR6ox2* is independent of BR signaling.

## Discussion

**FIGURE 4:**
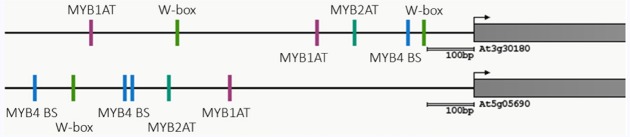
**Common transcription binding sites present in the promoters of ***CPD*** (At5g05690) and ***BR6ox2*** (At3g30180).** The figure has been modified from the Athena output (http://www.bioinformatics2.wsu.edu/cgi-bin/Athena/cgi/home.pl).

A crosstalk between flg22-triggered and BR signaling had long been postulated, given that both signaling pathways (i) lead to opposing outcomes (i.e., onset of defense versus activation of growth), and (ii) share components involved in signal initiation or transduction (Belkhadir et al., 2014; Lozano-Durán and Zipfel, 2015). Such an interaction was later experimentally confirmed and described to be negative, unidirectional (since only activation of BR signaling negatively affects PTI signaling, and not *vice-versa*), and at least partially indirect (Vert and Chory, 2011; Albrecht et al., 2012; Belkhadir et al., 2012; Lozano-Durán et al., 2013; Fan et al., 2014; Malinovsky et al., 2014). In these studies, however, activation of PTI signaling was achieved by exogenous flg22 treatment, within a time scale of min to very few hours, and therefore any potential longer-term effect of this pathway could have gone unnoticed. Additionally, activation of PTI could affect BR accumulation rather than sensitivity of the signaling pathway, which would be masked by exogenous hormone treatments or overexpression of rate-limiting components (Albrecht et al., 2012; Belkhadir et al., 2012). Our results indicate that, although activation of PTI signaling by flg22 has been shown not to affect BR signaling (Albrecht et al., 2012; Belkhadir et al., 2012), it leads to a repression of BR biosynthetic genes. This effect can be detected already 15 min after flg22 treatment, and is sustained during a 24-h treatment. The flg22-triggered transcriptional repression of the BR biosynthetic marker genes *CPD* and *BR6ox2* does not require BR perception or signaling, and therefore we hypothesize that it is a direct effect of the activation of flg22-induced PTI signaling. Since the promoters of *CPD* and *BR6ox2* contain binding sites for WRKY and MYB transcription factors (Figure 4; Athena; O’Connor et al., 2005), which are known to mediate defense responses, one hypothesis would be that PTI-activated transcription factors, such as the ones belonging to these families, may directly mediate repression of BR biosynthetic genes. This potential PTI-mediated repression of BR biosynthesis could serve a double purpose: on one hand, it would work to alleviate the BR-mediated repression of PTI upon detection of an impending pathogen; on the other, it would inhibit BR-mediated growth, hence contributing to redirect resources towards immunity and away from growth.

## Materials and Methods

### Plant Materials and Growth Conditions

*Arabidopsis thaliana* Col-0 was used as genetic background for all experiments. Seedlings were grown as described in Lozano-Durán et al., 2013. The mutant lines *bri1-301* and *bin2-1* and the transgenic line BZR1-YFP have been previously characterized (Gampala et al., 2007; Peng et al., 2008; Xu et al., 2008).

### RNA Extraction

RNA was extracted from 14-day-old seedlings as described in (Couto et al., 2015).

### Quantitative Real-time PCR

First-strand cDNA synthesis was performed with the SuperScript III RNA transcriptase (Invitrogen) and oligo(dT) primer, according to the manufacturer’s instructions. For qPCR reactions, the reaction mixture consisted of cDNA first-strand template, primers (10 pmol each) and SYBR Green JumpStart Taq ReadyMix (Sigma). qPCR was performed in a BioRad CFX96 real-time system. *UBQ10* was used as the internal control; expression in mock-treated Col-0 seedlings was used as the calibrator, with the expression level set to one. Relative expression was determined using the comparative Ct method (2-ΔΔCt). Each data point is the mean value of three biological replicates.

### Protein Extraction and Immunoblotting

Protein extraction from 14-day-old seedlings and immunoblotting were performed as described in (Albrecht et al., 2012).

### Conflict of Interest Statement

The authors declare that the research was conducted in the absence of any commercial or financial relationships that could be construed as a potential conflict of interest.
